# Analysis of Bacterial Community During Cow Manure and Wheat Straw Composting and the Isolation of Lignin-Degrading Bacteria from the Compost

**DOI:** 10.3390/microorganisms13081716

**Published:** 2025-07-22

**Authors:** Hanxiang Yang, Jianguo Hu, Bingxin Zhang, Yan Li, Chenxian Yang, Fusheng Chen, Tingwei Zhu, Ying Xin

**Affiliations:** College of Food Science and Engineering, Henan University of Technology, Zhengzhou 450001, China; 13103840110@163.com (H.Y.); 17324599323@163.com (J.H.); 18853654694@163.com (B.Z.); hcp011016@163.com (Y.L.); fushengc@haut.edu.cn (F.C.); zhutingwei@haut.edu.cn (T.Z.); huilier323@126.com (Y.X.)

**Keywords:** compost, bacterial community structure, lignin degradation, isolation, bacteria

## Abstract

Biodegradation is a green and efficient method for lignin depolymerization and conversion. In order to screen potential bacterial strains for efficient lignin degradation, composts of cow dung and wheat straw were prepared, and the dynamic changes in the predicted bacterial community structure and function in different periods of the composts were investigated. Then, bacteria with an efficient lignin degradation ability were finally screened out from the compost samples. Based on the monitoring results of the physicochemical indexes of the composting process, it was found that the temperature and pH of the compost firstly increased and then decreased with the extension of time, and the water content and C/N gradually decreased. High-throughput sequencing of compost samples from the initial (DA), high-temperature (DB), and cooling (DC) periods revealed that the number of OTUs increased sharply then stabilized around 2000, and the alpha diversity of the bacterial community decreased firstly and then increased. The predominant phyla identified included *Proteobacteria*, *Firmicutes*, *Chloroflexi*, and *Bacteroidetes*, determined by the relative abundance of beta-diversity-associated species. Functional gene analysis conducted using Tax4Fun revealed that the genes were primarily categorized into Metabolism, Genetic Information Processing, Environmental Information Processing, and Cellular Processes. Based on the decolorization of aniline blue and the degradation efficiency of alkali lignin, eight bacterial strains were isolated from compost samples at the three stages. *Cupriavidus* sp. F1 showed the highest degradation of alkali lignin with 66.01%. *Cupriavidus* sp. D8 showed the highest lignin degradation potential with all three enzyme activities significantly higher than the other strains. The results provide a strategy for the lignin degradation and utilization of biomass resources.

## 1. Introduction

Biomass resources are abundant, with about 700 million tons of straw produced annually in China. However, a significant portion remains underutilized, resulting in a considerable wastage of natural resources and environmental pollution [1,2,3]. Straw serves as a rich source of the biopolymer lignocellulose, primarily composed of cellulose, hemicellulose, lignin, pectin, and other trace substances [4,5]. Among these components, cellulose and hemicellulose contain substantial amounts of renewable polysaccharides, which can be used as primary raw materials for ethanol production to replace fossil fuels [6,7]. However, in industrial applications, cellulose is tightly encased by lignin, making it difficult to expose its crystalline structure, thereby significantly restricting cellulose production and utilization efficiency [8]. Consequently, the depolymerization and removal of lignin not only facilitate the efficient release of cellulose and hemicellulose [2,9], enhancing energy production efficiency and reducing dependence on fossil fuels, but also enable the conversion of lignin macromolecules into aromatic compound monomers. These monomers have applications in chemical, food, and pharmaceutical industries, contributing to an increase in the economic value of lignin [10,11,12].

Pre-treatment methods for lignocellulose include physical, chemical, and biological approaches [13,14,15,16,17,18]. However, physical and chemical methods often require costly equipment and may generate toxic and hazardous waste, whereas biological methods have become a primary focus for lignin degradation research due to their advantages, including low cost, environmental sustainability, and continuous production [2,19,20,21]. Among biological approaches, fungi were the first organisms extensively studied for lignin degradation [22,23]. However, fungal growth is constrained by stringent conditions, such as high pH, anoxic environments, and elevated substrate concentrations, which significantly hinder their development. Additionally, the metabolic engineering of fungi is challenging due to their complex genetic systems [24,25]. In comparison, bacteria have emerged as a promising alternative for lignin transformation and high-value utilization, owing to their strong adaptability, simpler gene expression systems, and ease of genetic manipulation [26,27]. In recent years, advancements in culture techniques and high-throughput sequencing technology have facilitated the identification of lignin-degrading bacteria from various habitats, including the ocean [28], soil [29,30,31,32,33,34], tobacco straw [35], bagasse [36], and oil palm empty fruit bunch [37]. These bacteria predominantly belong to *Actinobacteria*, *Proteobacteria*, and *Firmicutes* [38,39]. As herbivorous ruminants, cattle possess a diverse intestinal microbiota capable of degrading lignocellulose, making cow manure a potential source of lignocellulose-degrading bacteria. Composting, which involves mixing cow dung with crushed straw, is a dynamic process characterized by a complex bacterial community that undergoes structural changes throughout composting. High-throughput sequencing technology enables the analysis of bacterial diversity and community structural shifts during composting, facilitating the identification of dominant species at different stages and the isolation of strains with a high lignin degradation potential.

In this study, compost was prepared by thoroughly mixing cow manure with crushed wheat straw. The variations in physicochemical parameters during the composting process were monitored and analyzed, and compost samples from different stages were subjected to 16S high-throughput sequencing to examine the bacterial community structure based on changes in physicochemical indices during cow manure–wheat straw composting. The degradation rate of alkali lignin was measured, and bacterial strains with lignin degradation potential were isolated from composted materials at different stages. Additionally, the activities of lignin peroxidase, laccase, and manganese peroxidase were assessed to evaluate the lignin degradation capacity of the selected strains. The novelty of this study lies in directly linking the temporal metagenomic profiling of the composting microbiome with the concurrent isolation and efficiency screening of cultivable, high-performance ligninolytic strains from the same experimental process.

## 2. Materials and Methods

### 2.1. Composting Process and Sampling

The natural composting pile, consisting of cow manure, wheat straw, and corn straw, was prepared in Zhengzhou, Henan Province, China. The crushed wheat straw, corn straw, and cow manure were thoroughly mixed at a C:N ratio of 32:1. Then, sterilized ultrapure water was added in small amounts and multiple times using a sprayer. During turning and mixing, materials from top to bottom and inside to outside were made to make contact with water uniformly, adjusting the moisture content to approximately 70%. The mixtures were composted in insulated foam board containers with a volume of 200 L. Composting was conducted indoors, with the sample cultured under a constant temperature and in a humidity incubator at 37 °C for 11 days before being transferred to a laboratory room-temperature environment (25–30 °C) for cultivation until day 45. And the sample mixtures were turned four times on days 3, 6, 11, and 21 during the composting process. Samples were taken at 0, 1, 3, 5, 7, 9, 11, 13, 15, 17, 19, 23, 27, 29, 34, and 45 d. Samples were collected from different areas of the compost (upper, middle, and lower). Samples were taken from the left, middle, and right sections of the different areas and mixed thoroughly as a sample. This was repeated three times and collected as the representative sample of the day. The collected samples were divided into two portions: one was stored at 4 °C for physicochemical analysis, while the other was preserved at −80 °C for DNA extraction and microbial analysis.

### 2.2. Compost Physicochemical Properties Analysis

Temperature was monitored daily at the bottom, core, and surface of the composting pile using thermometers. Moisture content was determined by calculating mass loss after oven-drying at 105 °C. Samples were then mixed with deionized water at a 1:10 (*w*/*v*) ratio and shaken for 2 h, after which the pH was measured using a pH meter. Organic matter (OM) content was determined by the loss on ignition method in a muffle furnace at 550 °C, followed by the determination of the total organic carbon (TC) content. Total nitrogen [40] content was analyzed using the H_2_SO_4_-H_2_O_2_ digestion method on an Automatic Kjeldahl Analyzer [41,42].

### 2.3. DNA Extraction and High-Throughput Sequencing

Samples from the initial phase (day 0, DA), thermophilic phase (day 1, DB), and cooling phase (day 6, DC) were collected to analyze bacterial community composition using the Illumina MiSeq platform at Beijing Novogene Co. Ltd. (Beijing, China). Total bacterial genomic DNA was extracted from these samples using the DNA extraction kit from Tiangen Biotech (Beijing) Co., Ltd. (Beijing, China), and DNA integrity was assessed through 1% agarose gel electrophoresis. PCR amplification targeted the V3 and V4 hypervariable regions of bacterial 16S rRNA genes. The primer pairs 515F and 806R were used to amplify the V4 region of the bacterial 16S rRNA gene (515F: 5′-GTGCCAGCMGCCGCGGTAA-3′, 806R: 5′-GGACTACHVGGGTWTCTAAT-3′) [43,44,45]. Each PCR reaction was performed in a 30 µL volume containing 15 µL of Phusion^®^ High-Fidelity PCR Master Mix (New England Biolabs, Ipswich, MA, USA), 0.2 µM of forward and reverse primers, and about 10 ng of template DNA. The thermal cycling conditions included initial denaturation at 98 °C for 1 min, followed by 30 cycles of denaturation at 98 °C for 10 s, annealing at 50 °C for 30 s, and elongation at 72 °C for 30 s, with a final extension at 72 °C for 5 min. PCR products were mixed with an equal volume of 1X loading buffer containing SYB green and analyzed by 2% agarose gel electrophoresis. Aliquots were mixed according to the concentration of the PCR product. PCR products were pooled in equidensity ratios and purified using the Qiagen Gel Extraction Kit (Qiagen, Hilden, Germany). Sequencing libraries were prepared using the TruSeq^®^ DNA PCR-Free Sample Preparation Kit (Illumina, San Diego, CA, USA), and library quality was assessed using the Qubit@ 2.0 Fluorometer (Thermo Scientific, Waltham, MA, USA) and the Agilent Bioanalyzer 2100 system. Finally, sequencing was performed on an Illumina NovaSeq platform with a sequencing depth of 100,000 raw reads per sample, generating 250 bp paired-end reads.

### 2.4. Bioinformatics and Data Analysis

Raw sequence reads were filtered before subsequent analyses to minimize the effects of random sequencing errors. Raw tags were truncated at the first low-quality base (Phred score ≤ 19) initiating a stretch of ≥3 consecutive low-quality bases. Tags were then filtered to retain only those where the longest contiguous high-quality segment constituted ≥75% of the total length. The obtained tags were processed to remove chimeric sequences, the tags sequences were compared with the Species Annotation Database (https://github.com/torognes/vsearch/, accessed on 26 May 2023) to detect chimeric sequences, and the chimeric sequences were finally removed to obtain the final effective tags. About 65,000 reads per sample remained after quality filtering. Rarefaction was performed before diversity analysis. Uparse software (Uparse v7.0.1001, http://www.drive5.com/uparse/, accessed on 26 May 2023) was used to cluster all effective tags from the samples, with sequences grouped into Operational Taxonomic Units (OTUs) at a 97% similarity threshold. The sequences with the highest frequency within each OTU were selected as representative sequences for further analysis. Species annotation of OTU sequences was performed using the Mothur method with SSUrRNA data from SILVA138 (http://www.arb-silva.de/, accessed on 26 May 2023), applying a threshold range of 0.8 to 1, to obtain taxonomic information and determine the community composition at the phylum and class levels. Alpha-diversity indices, including Chao1 and Shannon indices, were calculated using Qiime software (Version 1.9.1), and species cumulative curves were generated. Between-group differences in alpha-diversity indices were analyzed using the Wilcox test in R software (Version 2.15.3). UniFrac distance was calculated, and an unweighted pair group method with an arithmetic mean (UPGMA) sample clustering tree was constructed using Qiime software (Version 1.9.1). Non-metric multidimensional scaling (NMDS) plots were generated in R software. Beta-diversity-index intergroup differences were analyzed using the MRPP function from the vegan software package (Version 2.15.3), with a Wilcox test performed based on the agricolae package. Tax4Fun functional prediction was conducted by extracting 16S rRNA gene sequences from the KEGG database prokaryotic genome and aligning them to the SILVA SSU Ref NR database (BLAST bitscore > 1500) (https://www.arb-silva.de/, accessed on 26 May 2023) using the BLASTN algorithm to establish a correlation matrix. Functional information from the KEGG database, annotated by UProC and PAUDA, was mapped to the SILVA database to achieve functional annotations. The sequenced samples were clustered into OTUs using the SILVA database sequences as reference sequences to obtain functional annotation information.

### 2.5. Isolation of Lignin-Degrading Bacteria from Compost

Compost samples (DA, DB, and DC) were collected and incubated in a selective medium containing lignin to isolate lignin-degrading bacteria. Alkali lignin was incorporated into the medium as the sole carbon and energy source, following the method described in a previous study [46]. The bacteria isolated from the composting pile were subsequently cultured on aniline blue agar plates to assess the decolorization zone, which served as an indicator of crude lignolytic enzyme activity [47].

### 2.6. 16S rRNA Sequencing Determination and Physiological Characteristics

Compost samples from days 0, 1, and 6 were selected for 16S amplicon high-throughput sequencing to analyze bacterial community structure changes and functional predictions. Genomic DNA of the isolated bacteria was extracted and purified using the Qiagen Gel Extraction Kit (Qiagen, Germany) for PCR amplification. The primers 27F (5′-AGAGTTTGATCCTGGCTCAG-3′) and 1492R (5′-GGTTACCTTGTTACGACTT-3′) were used to amplify the 16S rRNA gene. The PCR products were sequenced at Sangon Biotech Co., Ltd. (Shanghai, China). The obtained sequences were identified using the BLAST alignment search tool (https://blast.ncbi.nlm.nih.gov/Blast.cgi, accessed on 26 May 2023). A circular phylogenetic tree was constructed using MEGA 5.0 and visualized with iTOL (https://itol.embl.de/#, accessed on 26 May 2023).

### 2.7. Determination of the Biodegradation of Alkali Lignin

Biodegradation experiments were conducted to evaluate the lignolytic degradation ability of selected isolates. The isolates were inoculated (10%, *v*/*v*) into an alkali lignin medium containing (g/L) NaNO_3_, 2.5; MgSO_4_, 1.0; KH_2_PO_4_, 1.0; NaCl, 1.0; Na_2_HPO_4_, 1.0; NH_4_Cl, 1.0; and alkali lignin, 2.0. The cultures were incubated at 37 °C with shaking at 150 r/min for 7 days. Uninoculated samples were prepared as controls under the same conditions. After the degradation process, the residual alkali lignin in the culture supernatant was quantified by measuring absorbance at 280 nm [48,49,50].

### 2.8. Lignolytic Enzyme Activity

Lignolytic enzyme activity was determined by measuring the oxidation of three substrates—ABTS, 2,6-DMP, and veratryl alcohol—using a UV-VIS spectrophotometer. The oxidation of ABTS, 2,6-DMP, and veratryl alcohol was monitored at 420 nm (ε420 = 36,000 M^−1^ cm^−1^), 310 nm (ε310 = 9300 M^−1^ cm^−1^), and 470 nm (ε470 = 69,600 M^−1^ cm^−1^), respectively. One unit of enzyme activity (U) was defined as the amount of enzyme required to oxidize 1 µmol of substrate per minute [46].

### 2.9. Statistical Analysis

All experiments were conducted in triplicate, and the results are expressed as mean ± standard deviation. Analysis of variance [51] was performed to evaluate significant differences among all measured results using SPSS 20.0. A *p*-value of <0.05 was considered statistically significant.

## 3. Results

### 3.1. Changes in Physicochemical Parameters During Composting

**Figure 1:**
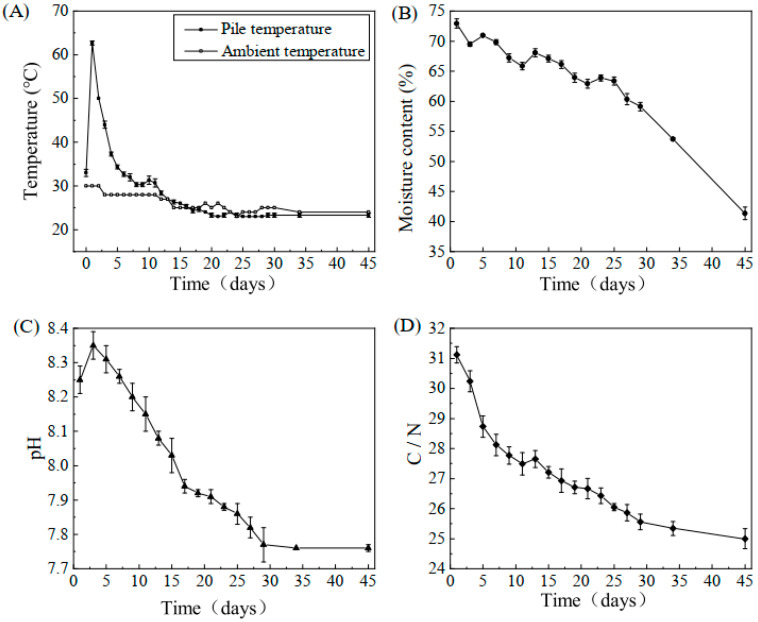
Changes in various physical and chemical indicators during composting. (**A**) Variations in pile temperature and ambient temperature throughout composting; (**B**) variations in moisture content during composting; (**C**) variations in pH during composting; (**D**) variations in C/N ratio during composting. Error bars represent standard deviation from the mean (n = 3).

The variations in physical and chemical parameters during the composting process are presented in Figure 1. Temperature fluctuations influence microbial growth and reproduction, as shown in Figure 1A. Based on the observed changes, the composting process can be divided into four distinct phases: the mesophilic stage (0–1 d), thermophilic stage (1–3 d), cooling stage (4–20 d), and maturity stage (21–45 d). At the beginning of composting, the initial temperature was 30 °C, which increased rapidly within the first day. The decomposition of easily degradable organic matter by microorganisms generated a significant amount of heat, raising the temperature to a peak of 62.7 °C, marking the onset of the thermophilic stage. The temperature remained above 40 °C for three days, during which lignocelluloses began to degrade, contributing to humus formation. In the later phase, as easily degradable organic matter was consumed, microbial degradation decreased, leading to a gradual decline in pile temperature, eventually reaching levels close to the ambient temperature.

The moisture content change curve is illustrated in Figure 1B. Throughout the process, a gradual decline in moisture content was observed due to microbial water consumption. As depicted in Figure 1B, the initial moisture content of 72.92% decreased to 41.35%. Temporary fluctuations and brief increases in moisture content were observed at days 3, 6, 11, and 21 due to pile turning, which ensured adequate aeration and oxygen supply.

The pH change curve is shown in Figure 1C. At the start of composting, nitrogen-containing organic matter decomposed rapidly, producing ammonia nitrogen. Simultaneously, a portion of organic acids was oxidized, decomposed, and volatilized, leading to a rapid increase in pH. As composting progressed, ammonia release decreased, and organic acids formed from macromolecular decomposition neutralized the pH, bringing it from 8.35 to 8.03. After the cooling phase, the decomposition of readily degradable organic matter was nearly complete, resulting in a weakly alkaline final compost that reached a stable maturity state.

Due to the continuous consumption of organic carbon, the C/N ratio exhibited a downward trend throughout the composting process. As shown in Figure 1D, the initial C/N ratio of 31.12 decreased to 25.00 at the maturity stage. A rapid decline was observed in the first five days, attributed to the accelerated microbial activity utilizing easily degradable organic matter, leading to a sharp reduction in carbon content. Subsequently, as the availability of degradable substances decreased, microbial activity declined, causing a slower reduction in the C/N ratio.

### 3.2. 16S Amplicon High-Throughput Sequencing

**Figure 2:**
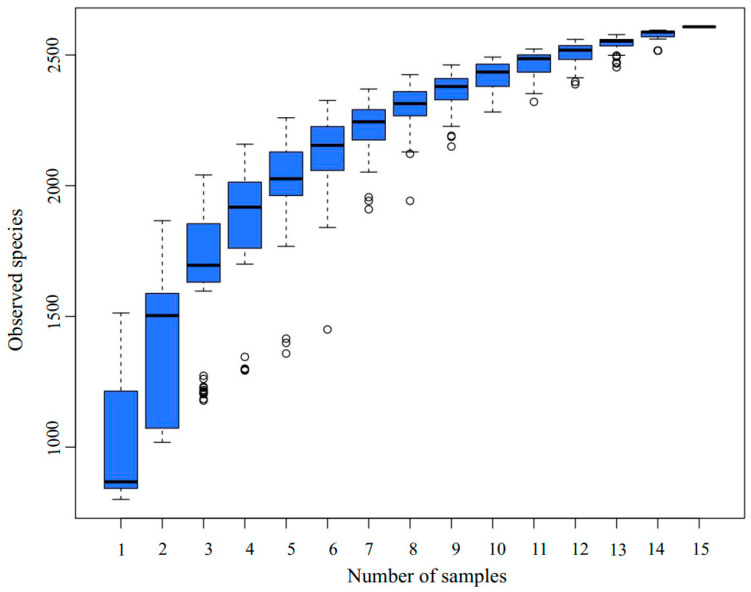
Box plot of species accumulation.

**Figure 3:**
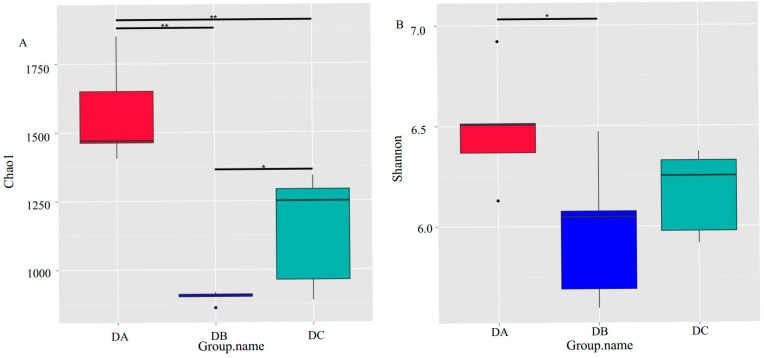
Alpha diversity of the bacterial community during composting. (**A**) Chao1 index; (**B**) Shannon index. *p*-values were determined using Wilcox signed-rank tests, * *p* < 0.05, ** *p* < 0.01.

A total of 15 samples were selected for 16S amplicon high-throughput sequencing at three time points, day 0 (DA), day 1 [52], and day 6 (DC), based on the variations in the physical and chemical parameters of the composting pile. The emergence rate of new OTUs (new species) under continuous sampling is illustrated in Figure 2. As observed in the figure, the position of the box plot initially increases sharply with the expansion of the sample size, indicating the identification of a substantial number of species within the community. Subsequently, the position of the box plot stabilized at around 2500, suggesting that the sequencing depth was sufficient and most OTUs in the compost samples were captured within the available sequencing volume [53]. The alpha diversity of the bacterial community throughout the composting process is depicted in Figure 3. The Chao1 index (*p* = 0) (Figure 3A) and Shannon index (*p* = 0.0153) (Figure 3B) show a significant decline. During the DC phase, the Chao1 index exhibits a significant increase (*p* = 0.0377), whereas the Shannon index increases, but not to a statistically significant extent.

### 3.3. Evolution of Bacterial Community During Composting

**Figure 4:**
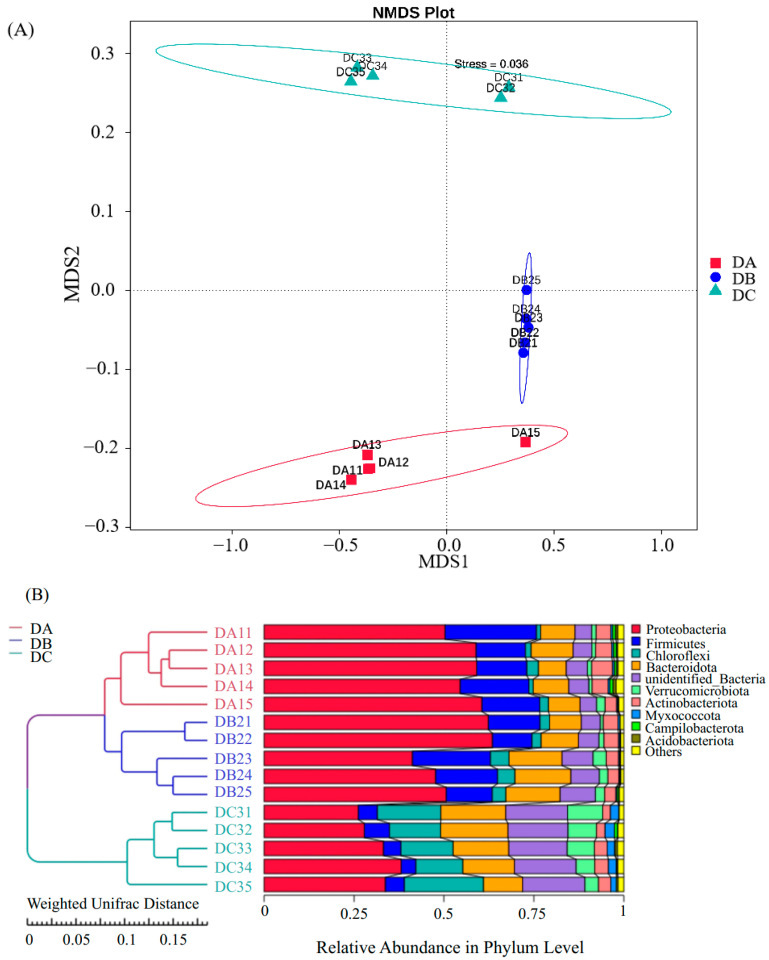
Community distribution and species composition across different samples. (**A**) NMDS analysis results at the OTU level. (**B**) UPGMA clustering tree based on weighted UniFrac distance, combined with phylum-level relative abundance distribution. Stress < 0.2, indicating that NMDS accurately represents the degree of variation between samples.

The Multi-Response Permutation Procedure (MRPP) analysis of beta diversity, based on Non-Metric Multi-Dimensional Scaling (NMDS) and the weighted UniFrac distance, indicated that the bacterial community structure among the three groups (DA, DB, and DC) exhibited greater differences between groups than within groups, with statistically significant differences (*p* < 0.05) (Table S1). NMDS analysis at the OTU level (Figure 4A) demonstrates that DA, DB, and DC occupy distinct clusters, although DA and DB are positioned relatively close, indicating minimal variation between these two groups. *Firmicutes* was the most abundant phylum during the entire composting process (DA: 56.72%; DB: 53.12%; DC: 1.85%). As composting progressed, the relative abundances of *Proteobacteria* and *Firmicutes* declined by 24.87% and 12.32%, respectively (Table S2). In contrast, *Chloroflexi*, *Bacteroidota*, and *Verrucomicrobiota* exhibited a gradual increase and were more evenly distributed in DC, with *Chloroflexi* showing a 14.35% increase.

### 3.4. Function Prediction

**Figure 5:**
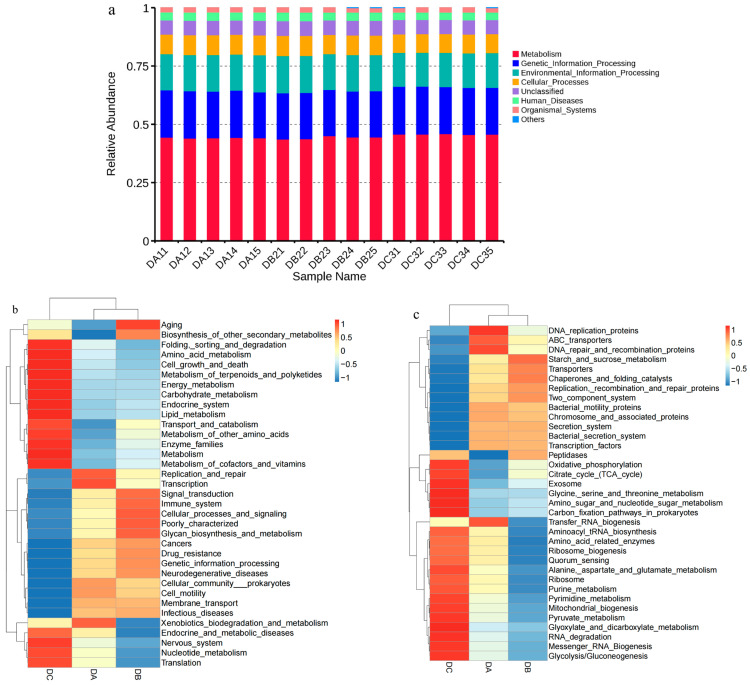
Tax4Fun gene function annotation of compost samples. (**a**) Level-1 functional categories; (**b**) level-2 functional categories; (**c**) level-3 functional categories.

The functional characteristics of bacterial communities in compost were predicted using Tax4Fun based on the KEGG database. The relative abundance of various functional genes within and between groups showed minor variations. Most functional genes were classified into Metabolism (43.65–45.98%), Genetic Information Processing (19.65–20.52%), Environmental Information Processing (14.54–15.94%), and Cellular Processes (7.91–8.60%) (Figure 5a). During the composting process, the relative abundances of Carbohydrate Metabolism, Amino Acid Metabolism, and Lipid Metabolism increased from 9.30%, 9.05%, and 2.96% to 9.67%, 9.32%, and 3.26%, respectively (Figure 5b). Specifically, the relative abundances of Amino Sugar and Nucleotide Sugar Metabolism, Carbon Fixation Pathways in Prokaryotes, Oxidative Phosphorylation, and Pyruvate Metabolism showed an increasing trend (Figure 5c), likely due to the easier degradation of organic compounds, such as proteins and lipids during composting. Translation activity initially declined from 7.89% to 7.70% before increasing to 8.13%, primarily attributed to the enhancement of RNA degradation and Ribosome Biogenesis (Figure 5b,c). This fluctuation may be due to the inhibitory effect of high temperatures during DB on bacterial reproduction, followed by recovery as the temperature declined. Conversely, Membrane Transport and Cell Motility decreased from 11.27% and 3.04% to 10.49% and 2.52%, respectively, mainly reflected by the reduction in Replication, Recombination and Repair Proteins, Bacterial Motility Proteins, Secretion System, and Bacterial Secretion System activities (Figure 5b,c).

### 3.5. Screening of High-Efficiency Lignin-Degrading Strains

Alkali lignin was used as the sole carbon source in the medium to isolate lignin-degrading bacteria from compost samples collected on days 0, 1, and 6. After 14 days of co-cultivation, 115 bacterial strains were selected for further isolation and purification. A total of 64 strains were obtained from the day 0 sample, designated as A1–A8, B1–B6, C1–C30, D1–D15, and F1–F5; 30 strains from the day 1 sample, numbered K1–K30; and 21 strains from the day 6 sample, labeled L1–L21. Therefore, 110 strains obtained after continuous streaking, isolation, and purification were introduced into perforated aniline blue solid medium, and the lignin degradation ability was assessed based on the size of the decolorization zone around the bacterial culture.

**Figure 6:**
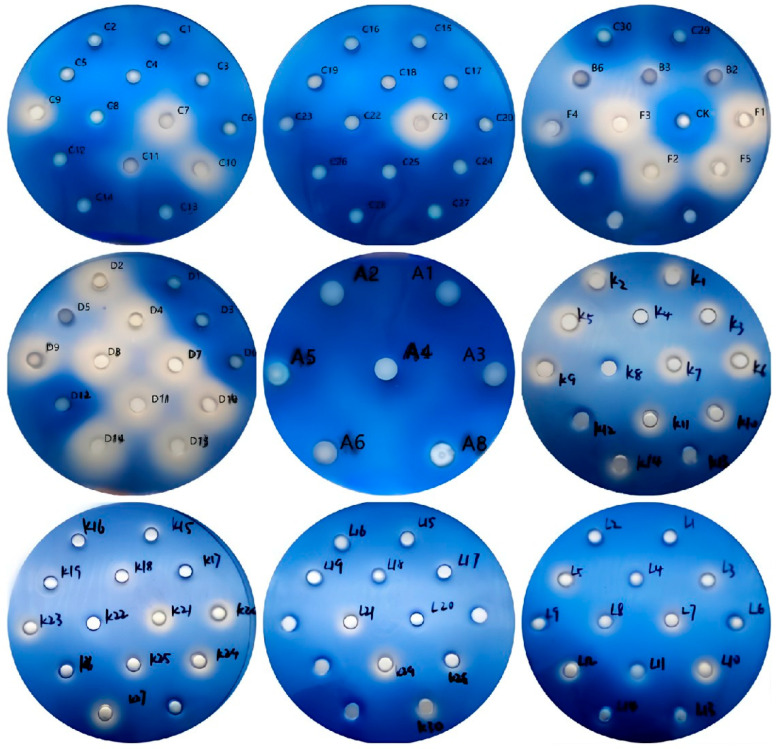
Decolorization of aniline blue by the selected strains.

The decolorization effect of the 110 bacterial strains on aniline blue is presented in Figure 6. The strains at different composting stages exhibited varying degrees of decolorization. Among the 64 strains obtained from the day 0 compost sample, 16 strains (C7, C21, F1–F3, F5, D2, D4, D7–D11, D13, and D14) demonstrated a pronounced decolorization effect. Some strains, including C9-C11, C30, B2, B3, B6, F4, D5, and A5, exhibited decolorization, though the effect was less pronounced. The remaining 38 strains did not decolorize aniline blue. In the day 1 compost sample, 30 strains were isolated, of which 23 (K1–K7, K9–K11, K14, K15, K18, K20, K21, and K23–K30) exhibited weak decolorization, while the remaining 7 strains showed no decolorization. In the day 6 compost sample, 8 of the 21 strains (L3, L5, L7, L8, L10–L12, and L21) demonstrated slight decolorization, whereas the remaining 13 strains did not decolorize aniline blue.

### 3.6. Degradation of Alkali Lignin by Lignin-Degrading Strains

**Figure 7:**
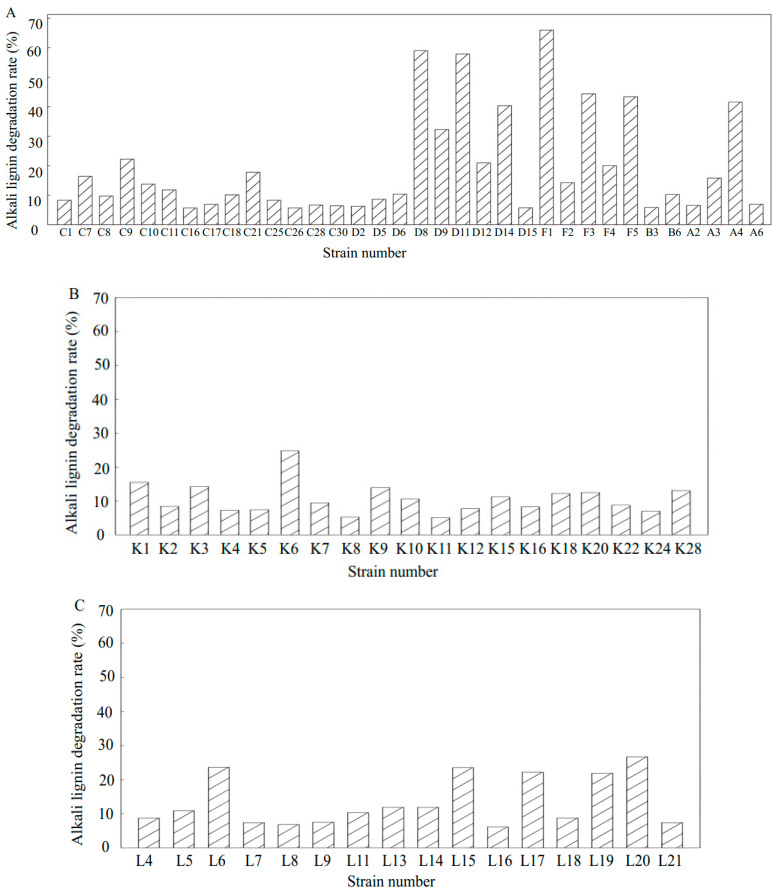
Biodegradation of alkali lignin by strains at different composting stages over 7 days. (**A**) Strains selected from the initial stage of composting; (**B**) strains selected from the high-temperature phase of composting; (**C**) strains selected from the compost cooling phase. (Strains with an alkali lignin degradation rate below 5% were excluded from the analysis).

To further evaluate the lignin degradation potential of the 110 selected strains, the activated culture solutions of these strains were inoculated into a medium containing 1 g/L alkali lignin and incubated for 7 days. The residual alkali lignin content was measured at 280 nm using an ultraviolet spectrophotometer, and the degradation rate was calculated using a degradation medium without bacterial inoculation as a blank control. The results are presented in Figure 7. Among the 64 strains isolated from the day 0 compost sample, six strains (D8, D11, F1, F3, F5, and A4) exhibited a higher alkali lignin degradation rate. In the day 1 group, two strains (K1, K6) demonstrated a high degradation rate of alkali lignin. From the 21 strains obtained from the day 6 compost sample, 6 strains (L6, L15, L17, L19, L20, and L21) displayed a higher alkali lignin degradation rate. Based on the results of aniline blue decolorization, seven strains (K6, L17, L20, L21, D8, D11, and F1) were selected for subsequent experiments.

### 3.7. Identification of Isolated Lignin-Degrading Strains

**Figure 8:**
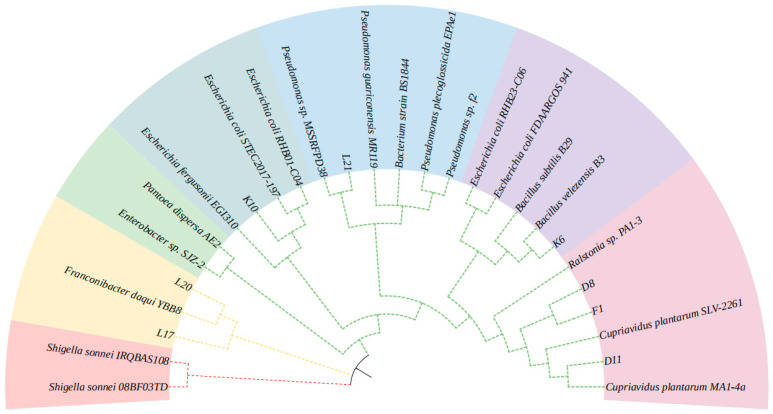
Phylogenetic tree of the isolated potential bacterial strains.

The sequencing results of the isolated strains obtained from the secondary screening were compared with the BLAST column similarity in the NCBI database. The homology similarity between each strain and its corresponding reference strain exceeded 98%. Based on the 16S rDNA sequence analysis, a phylogenetic tree was constructed, as shown in Figure 8. According to the BLAST sequence similarity comparison and phylogenetic tree homology analysis, strains D8, D11, and F1 were identified as members of the genus *Cupriavidus*, strain K6 belonged to the genus *Bacillus*, strain K10 was classified under the genus *Escherichia*, strains L17 and L20 were identified as *Franconibacter*, and strain L21 was categorised under the genus *Pseudomonas*. Among these strains, all except L21, which belongs to the *Firmicutes phylum*, were classified under *Proteobacteria*. This distribution pattern aligns with the relative abundance of species at the phylum level observed in different composting samples from previous 16S amplicon high-throughput sequencing. These findings further confirm that *Proteobacteria* and *Firmicutes* are dominant bacterial phyla with significant lignin degradation capabilities in compost utilizing wheat straw as the sole carbon source.

### 3.8. Enzyme Activity of Isolated Lignin-Degrading Strains

**Figure 9:**
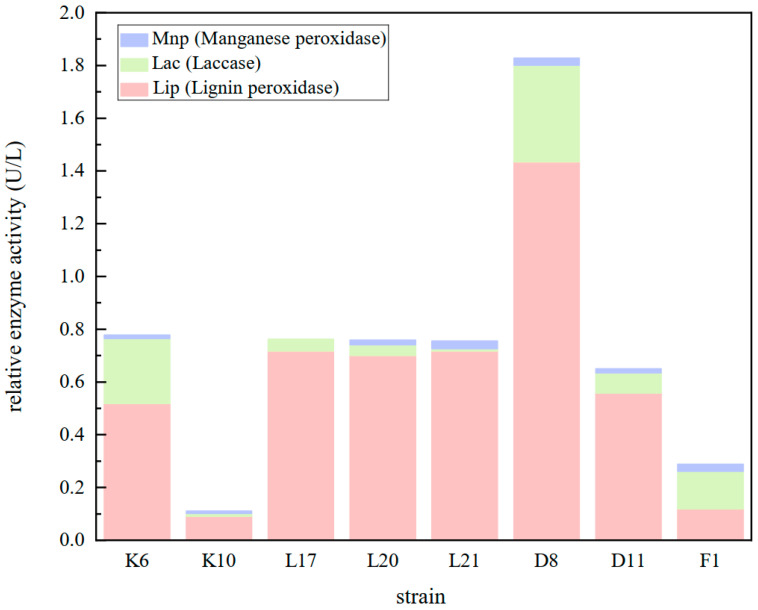
Comparison of relative enzyme activity results for the selected strains.

The microbial degradation of lignin primarily occurs through enzymatic reactions, where lignin peroxidase or laccase catalyzes oxidative degradation using hydrogen peroxide or molecular oxygen as an electron acceptor to break down the aromatic unit structure. The most widely reported lignin-degrading enzymes include lignin peroxidase (LiP), manganese peroxidase (MnP), and laccase (Lac). Therefore, the enzyme activity of the eight strains obtained from the re-screening was evaluated by monitoring the secretion of these three enzymes after 48 h of bacterial growth. As shown in Figure 9, all eight potential bacterial strains exhibited high lignin peroxidase activity, while manganese peroxidase and laccase activities were relatively low. Among these strains, MPD8 demonstrated the highest lignin peroxidase activity at 1.434 ± 0.056 U/L, whereas K10 and F1 exhibited the lowest enzyme activities, both below 0.20 U/L. The LiP activities of K6, L17, L20, and L21 were comparable, each measuring around 0.60 U/L. Regarding manganese peroxidase activity, except for L17, which did not produce MnP, L21, D8, and F1 exhibited higher MnP activities, whereas the other three strains had lower MnP activity. Additionally, D8 and K6 displayed higher laccase activities, while the remaining six strains exhibited weak laccase activity. These findings indicate that lignin-degrading bacteria can secrete lignin-degrading enzymes but does not necessarily produce all types of these enzymes. Based on the overall performance of the three lignin-degrading enzymes, *Cupriavidus* sp. D8 was identified as the dominant strain for the further characterization of lignin degradation potential.

## 4. Discussion

The results demonstrate that the microbial succession driving cow manure–straw compost maturation is fundamentally shaped by the need to decompose recalcitrant polymers, like lignocellulose. By measuring various physicochemical indicators of compost samples, we evaluated the composting effect and selected suitable samples for analysis of microbial species distribution and changes in bacterial community structure within the compost heap. Temperature is a crucial indicator for assessing the maturation of the composting system. In our experiments, the compost temperature initially rose and then continued to increase. The temperature remained above 40 °C for 3 days. Similar results were observed by Meng et al. [54]. At this stage, lignocelluloses began degrading to form humus. Sundberg et al. [55] showed that lignin in the compost had started to be degraded during this period and most of the harmful bacteria and parasites were also inactivated during the high-temperature period of composting. In the later stages of composting, as a significant amount of easily degradable organic matter was consumed, the activity of the microorganisms lowered the temperature of the pile, making it similar to the ambient temperature during the decomposition period [56,57]. As microbial growth and metabolism depend on the solubility and bioavailability of organic matter, moisture content governs both its transfer within the compost pile and microbial activity, making it a critical factor in composting. In this study, the moisture content consistently decreased as the microorganisms utilized the water [58,59]. Similar findings were reported by Liu et al. [60] in a study of cattle manure composted with tobacco straw. Jain et al. [61] also obtained similar findings in their study of composting aquatic weeds, cow manure, and wood chips, and Zhong et al. [62] also observed similar patterns of moisture content changes in dairy manure. Additionally, Zhao et al. [63] studied the evolution of physical and chemical properties during the 45-day sheep manure composting process and observed similar trends in moisture content changes. During the composting process, the moisture content showed a continuous decreasing trend. While the bacteria in the compost heap performed metabolic activities to utilize the water, the heat generated by microbial metabolism accelerated water evaporation, leading to a decrease in the moisture content [64]. However, the water content in the piles was momentarily elevated because the pile was turned at 3 days, 6 days, 11 days, and 21 days to promote ventilation and oxygen supply. Changes in pile pH are closely associated with ammoniacal nitrogen. The optimal pH range for most microorganisms in composting is neutral to slightly alkaline [65,66]. Unsuitable pH conditions can decrease or even inhibit microbial activity, leading to slow compost degradation [67]. The pH value increased at the beginning of composting in this study, which may be attributed to ammonia production through ammonification and the breakdown of organic acids. As composting progressed, the pH decreased from 8.35 to 8.03. This decrease can be attributed to the production and accumulation of organic acids from the organic substrates [68,69]. By the end of the composting process, the compost was weakly alkaline and had nearly reached maturity. Meng et al. [54] studied the microbial population dynamics of dairy manure and rice straw during static (non-rotational) composting and found that the pH value in the heap tended to increase and then decrease (7.9 → 8.7 → 7.7), which is consistent with the results of this paper. During composting, the rate of decomposition of organic matter by microorganisms varied with the carbon-to-nitrogen (C/N) ratio [70]. An initially high or low C/N ratio in the compost material hindered the growth and reproduction of microorganisms as well as the utilization of organic matter [71,72]. A C/N ratio in the range of 26 to 35:1 is more appropriate. Due to the continuous consumption of organic carbon during the composting process, the C/N of the pile consistently showed a downward trend [73]. In the first five days, the C/N ratio declined quickly due to a rapid decrease in carbon content resulting from the microbial utilization of organic matter [74]. After this, the reduction in readily degradable substances led to decreased microbial activity and a slower drop in C/N values. In the later stages, the C/N ratio remained relatively stable because it became more challenging for microorganisms to utilize organic matter. In the final stage, humus formation and a decrease in pile temperature further limited microbial utilization of organic materials, stabilizing the C/N ratio.

The bacterial community structure in the pile at different stages of the composting process is different [65]. Analysis of alpha diversity revealed a significant decline in bacterial species richness and diversity in DB, as indicated by a decrease in Chao1 (*p* = 0) and Shannon indices (*p* = 0.0153). This reduction was likely due to high temperatures (62.4 °C), which eliminated heat-sensitive species, demonstrating the composting system’s self-purification mechanism. During DC, Chao1 increased significantly (*p* = 0.0377), while the Shannon index showed a slight but non-significant increase, suggesting that the initial microbial community included non-beneficial species, while high-temperature composting favored functional communities involved in organic matter degradation. These alpha-diversity trends were consistent with previous studies on straw and sewage sludge composting. With the extension of composting time, OTUs first decreased and then increased, and the bacteria of *Firmicutes* also gradually decreased, and the change pattern of the abundance of the bacterial community was the same as our study [75,76].

Sample similarity analysis was conducted using a weighted UniFrac distance matrix to construct a clustering tree, integrating taxonomic composition at the phylum level. Different groups clustered distinctly, with DA and DB forming sub-cluster I and DC forming sub-cluster II. The similarity between DA and DB indicated comparable species composition, whereas DC showed lower similarity. Similar findings were reported in previous studies [75,77]. Among all identified bacterial phyla in the composting samples, *Proteobacteria*, *Firmicutes*, *Chloroflexi*, and *Bacteroidetes* were the most dominant [78]. *Firmicutes* was the most abundant phylum throughout composting, as previously observed in plant waste composting. In this study, the relative abundance of *Chloroflexi* increased by 14.35% in DC, possibly due to its adaptation to high-temperature composting conditions. The variation in different bacterial occupancy ratios indicated that the environmental conditions of composting (temperature, C/N ratio, and water content) limitations influenced the changes in the microbial community structure of the compost, while Chloroflexi, Bacteroidetes and Verrucomicrobiota were more adapted to the compost environment. For example, Verrucomicrobiota plays an important role in the curing phase of decomposing organic materials, inducing lignin hydrolase production by microorganisms in organic materials and promoting lignin degradation [79]. Ultimately, the more uniform distribution of heap species at DC produced a more homogenized bacterial community composition compared to the initial community structure and composition of the compost. As a result, the bacterial community structure evolved until a unique microbial population structure emerged that was able to adapt to lignocellulose degradation. According to the Tax4Fun gene function annotation results, the relative abundance of carbohydrate metabolism, amino acid metabolism, and lipid metabolism increased. Specifically, the relative abundance of functional genes related to amino and nucleotide sugar metabolism, prokaryotic carbon fixation pathway, oxidative phosphorylation, and pyruvate metabolism were mainly increased. This might be due to the fact that when carbohydrates were metabolized under aerobic conditions during composting, various compounds were usually produced along with the degradation of readily degradable substances, such as lignocellulose, and then more complex molecules (i.e., lignin) were continuously utilized as energy substances for growth and reproduction [79]. Thus, carbohydrate metabolism played an important role in the degradation process of lignin. While Tax4Fun provided initial insights into potential functional shifts during composting, its limitations necessitate caution. Functional predictions derived solely from 16S data are constrained by database gaps, genetic heterogeneity within taxa, and an inability to capture regulatory dynamics. Metagenomic validation is therefore essential to confirm the presence of ligninolytic genes (e.g., MnP, LiP).

Preliminary and secondary screenings of compost samples from different time points (d0, d1, d6) enabled the selection of lignin-degrading bacteria. Since lignin-degrading enzymes, such as manganese peroxidase and lignin peroxidase, can be secreted by lignin-degrading strains, these enzymes oxidize aniline blue, leading to decolorization. Based on the ability to grow using alkali lignin and the decolorization of aniline blue, 110 bacterial strains were identified. Measurement of alkali lignin degradation rates led to the selection of eight strains with significant degradation capabilities: K6, K10, L17, L20, L21, D8, D11, and F1. Phylogenetic analysis revealed that all strains, except K6 (*Firmicutes*), belonged to the *Proteobacteria* phylum. Among them, *Cupriavidus* sp. D8 exhibited the highest alkali lignin degradation rate (59.05% in three days) and produced a prominent decolorization zone on aniline blue plates. The combined enzymatic activities of Lac, LiP, and MnP in D8 were higher than those in the other seven strains, leading to its selection as the dominant strain for further lignin degradation studies.

The isolated *Cupriavidus* sp. D8 demonstrated exceptional lignin degradation, showing high potential for applications in multiple fields, such as accelerating agricultural waste composting and the eco-friendly pre-treatment of lignocellulose in biorefineries. Next, we will analyze its enzymatic mechanism transcriptomics to confirm its application value in biomass resource utilization.

## 5. Conclusions

This study demonstrated that composting systems served as effective platforms for isolating lignin-degrading bacteria. Monitoring physicochemical parameters revealed the following characteristic composting dynamics: temperature and pH exhibited initial increased followed by declines, while moisture content and C/N ratio progressively decreased. High-throughput sequencing results documented significant microbial succession: alpha diversity showed an initial decrease followed by recovery. Functional prediction via Tax4Fun indicated predominant gene categorization within Metabolism, Genetic Information Processing, Environmental Information Processing, and Cellular Processes. Crucially, eight bacterial strains exhibiting ligninolytic potential were isolated based on aniline blue decolorization and alkali lignin degradation efficiency. Among these, while *Cupriavidus* sp. D8 displayed superior lignin degradation potential, these findings establish a strategic framework for harnessing microbial resources in composting to advance the sustainable degradation and valorization of lignin-rich biomass.

## Data Availability

The original contributions presented in this study are included in the article/Supplementary Materials. Further inquiries can be directed to the corresponding author.

## Supplementary Materials

The following supporting information can be downloaded at: https://www.mdpi.com/article/10.3390/microorganisms13081716/s1, Table S1: Analysis of differences between MRPP groups; Table S2: Distribution of top ten species at phylum level in different compost samples; Table S3: The important parameters of the sequencing and analysis process; Table S4: The OTU numbers of different samples; Table S5: The alkali lignin degradation rate and the decolorization zone size of isolated strains; Table S6: The enzyme activities of the selected strains.
