# Supplementary material for: Analysis of Bacterial Community During Cow Manure and Wheat Straw Composting and the Isolation of Lignin-Degrading Bacteria from the Compost
Source: Microorganisms. 2025 Jul 22;13(8):1716. doi: 10.3390/microorganisms13081716 (PMC12388791; doi:10.3390/microorganisms13081716)
Supplement: Supplementary file 1 [file microorganisms-13-01716-s001.zip › microorganisms-3682701-supplementary.pdf]

Table S1 Analysis of differences between MRPP groups

Group	A	Observed-delta	Expected-delta	Significance
DA-DC	0.4728	0.2325	0.441	0.008
DA-DB	0.1819	0.2625	0.3209	0.009
DB-DC	0.3923	0.2323	0.3822	0.011

Table S2 Distribution of top ten species at phylum level in different compost samples

Group	Proteobacteria	Firmicutes	Chloroflexi	Bacteroidota	Unidentified Bacteria	Verrucomicrobiota	Actinobacteriota	Myxococcota	Campilobacterota	Acidobacteriota	Others
DA	56.72%	17.63%	1.93%	9.50%	5.20%	1.34%	4.39%	0.48%	0.67%	0.42%	1.72%
DB	53.12%	15.40%	3.79%	12.89%	7.48%	2.16%	3.54%	0.42%	0.20%	0.02%	0.98%
DC	31.85%	5.31%	16.28%	15.46%	16.92%	6.84%	3.05%	2.18%	0.20%	0.32%	1.59%

Table S3 The important parameters of the sequencing and analysis process.

Sample_name	Total_reads	Combined_reads	Uncombined_reads	Percent_combined(%)	Combined_base(bp)	Min_len(bp)	Max_len(bp)	Avg_len(bp)
DA11	103,429	102,276	1,153	98.89	42,828,789	64	430	419
DA12	98,332	97,458	874	99.11	40,772,285	44	430	418
DA13	101,970	100,692	1,278	98.75	42,060,471	240	430	418
DA14	106,601	105,388	1,213	98.86	44,030,231	64	430	418
DA15	100,658	98,417	2,241	97.77	41,099,305	64	430	418
DB21	94,289	93,476	813	99.14	39,306,827	64	430	421
DB22	91,849	90,944	905	99.01	38,249,257	101	430	421
DB23	103,283	102,338	945	99.09	43,058,242	116	430	421
DB24	103,081	102,161	920	99.11	43,018,218	116	430	421
DB25	103,211	102,211	1,000	99.03	43,050,912	64	430	421
DC31	106,412	105,126	1,286	98.79	43,985,822	64	430	418
DC32	106,410	105,293	1,117	98.95	44,109,164	64	430	419
DC33	97,208	96,094	1,114	98.85	40,240,037	64	430	419
DC34	103,289	102,118	1,171	98.87	42,771,359	248	430	419
DC35	99,087	97,928	1,159	98.83	40,805,755	239	430	417
#Total	1,519,109	1,501,920	17,189	98.87	629,386,674	248	430	419

Table S4 The OTU numbers of different samples

Sample_name	OTUs
DA	2222
DB	1324
DC	2009

Table S5 The alkali lignin degradation rate and the decolorization zone size of isolated strains

Source	Strain	Alkali lignin degradation rate (%)	Decolorization zone size (R/mm)
DA	C1	9.52	—
	C7	16.41	1.8
	C8	9.77	1.2
	C9	22.26	2.2
	C10	13.75	2.2
	C11	12.97	—
	C16	5.66	—
	C17	6.87	—
	C18	10.13	—
	C21	17.80	3.0
	C25	8.32	—
	C26	6.51	—
	C28	6.69	—
	C30	6.45	1.30
	D2	6.26	3.30

D5	8.62	3.4
D6	10.37	1.3
D8	59.05	4.00
D9	32.29	3.80
D11	57.88	3.70
D12	20.94	3.80
D14	40.38	4.00
D15	5.72	3.80
F1	66.01	—
F2	14.23	3.4
F3	44.36	4.2
F4	20.03	3.8
F5	65.10	3.8
B3	5.84	1.40
B6	10.25	1.80
A2	5.96	—
A3	15.80	—
A4	41.58	—
A6	6.93	1.20

DB	K1	14.71	
	K2	8.32	1.60
	K3	14.03	1.10
	K4	7.35	0.41
	K5	7.35	2.30
	K6	24.63	4.10
	K7	9.14	3.80
	K8	5.47	—
	K9	13.98	1.80
	K10	23.75	1.60
	K11	5.22	1.55
	K12	7.40	—
	K15	11.18	—
	K16	8.42	—
	K18	12.19	0.80
	K20	12.43	—
	K22	8.61	—
	K24	7.11	0.8

	K28	13.01	—
DC	L4	8.61	—
	L5	10.89	1.30
	L6	17.75	—
	L7	7.21	1.10
	L8	6.87	—
	L9	7.40	—
	L11	9.82	0.50
	L13	12.43	—
	L14	11.76	—
	L15	7.40	—
	L16	5.90	—
	L17	22.06	—
	L18	8.08	—
	L19	8.42	—
	L20	26.22	—
	L21	22.79	—

Table S6 The enzyme activities of the selected strains

Strain	LiP(U/L)	Lac(U/L)	MnP(U/L)
K6	0.518	0.247	0.013
K10	0.09	0.012	0.009
L17	0.717	0.046	0
L20	0.7	0.041	0.018
L21	0.717	0.009	0.03
D8	1.434	0.367	0.027
D11	0.558	0.077	0.016
F1	0.119	0.142	0.027
